# Role of Cancer Stem Cells in Cholangiocarcinoma and Therapeutic Implications

**DOI:** 10.3390/ijms20174154

**Published:** 2019-08-25

**Authors:** Hsing-Ju Wu, Pei-Yi Chu

**Affiliations:** 1 Research Assistant Center, Show Chwan Memorial Hospital, Changhua 500, Taiwan; 2 Department of Medical Research, Chang Bing Show Chwan Memorial Hospital, Lukang Town, Changhua County 505, Taiwan; 3 Graduate Institute of Biomedical Engineering, National Chung Hsing University, Taichung 402, Taiwan; 4 School of Medicine, College of Medicine, Fu Jen Catholic University, New Taipei City 231, Taiwan; 5 Department of Pathology, Show Chwan Memorial Hospital, Changhua 500, Taiwan; 6 Department of Health Food, Chung Chou University of Science and Technology, Changhua 510, Taiwan

**Keywords:** cholangiocarcinoma, cancer stem cells, surface markers, tumor microenvironment, epithelial-to-mesenchymal transition, targeted therapy

## Abstract

Cholangiocarcinoma (CCA) is the second most common type of liver cancer, and is highly aggressive with very poor prognosis. CCA is classified into intrahepatic cholangiocarcinoma (iCCA) and extra-hepatic cholangiocarcinoma (eCCA), which is further stratified into perihilar (pCCA) and distal (dCCA). Cancer stem cells (CSCs) are a subpopulation of cancer cells capable of tumor initiation and malignant growth, and are also responsible for chemoresistance. Thus, CSCs play an important role in CCA carcinogenesis. Surface markers such as CD133, CD24, CD44, EpCAM, Sox2, CD49f, and CD117 are important for identifying and isolating CCA CSCs. CSCs are present in the tumor microenvironment (TME), termed ‘CSC niche’, where cellular components and soluble factors interact to promote tumor initiation. Epithelial-to-mesenchymal transition (EMT) is another important mechanism underlying carcinogenesis, involved in the invasiveness, metastasis and chemoresistance of cancer. It has been demonstrated that EMT plays a critical role in generating CSCs. Therapies targeting the surface markers and signaling pathways of CCA CSCs, proteins involved in TME, and immune checkpoint proteins are currently under investigation. Therefore, this review focuses on recent studies on the roles of CSCs in CCA; the possible therapeutic strategies targeting CSCs of CCA are also discussed.

## 1. Introduction

Hepatocellular carcinoma (HCC) and cholangiocarcinoma (CCA) are the first and second most common types of liver cancer, respectively [1,2,3]. CCA was first identified by Steiner and Higginson [4,5], and is highly aggressive with very poor prognosis. CCA is one of the most difficult intra-abdominal cancers, occurring via the malignant transformation of the epithelium lining of the biliary tree, called cholangiocytes, that derive from the bile ductules. CCA is a highly heterogeneous tumor in terms of anatomical location, pathology, and clinical features. The second-order bile ducts are used to classify intrahepatic cholangiocarcinoma (iCCA) and extra-hepatic cholangiocarcinoma (eCCA). One-third of CCA is iCCA, which derives from the parenchyma of the liver, while two-thirds of CCA is eCCA, which originates from the hepatoduodenal ligament within the biliary tree [2,6,7,8].

The incidence of CCA is the highest in northeast Thailand, approximately 100 per 100,000 people for men and 50 per 100,000 people for women [9]; however, its incidence is much lower in the Western world occurring in 1–2 cases per 100,000 resulting from the different risk factors relating to different ethnic, genetic, and environmental backgrounds [10,11]. Several epidemiological studies have revealed an increase in its global incidence (up to 10 folds) and mortality for iCCA, whereas that for eCCA either remained stable or slightly decreased [12,13,14]. A previous report demonstrated that the 5-year and 10-year survival rates are 32.3% and 8.4% for iCCA patients following resection, respectively [15]. Although CCA is a very aggressive cancer, it has not been studied as extensively as HCC.

eCCA can be further stratified into perihilar (pCCA) (also called Klatskin tumor) and distal (dCCA), based on its location in the mid or distal part of the main bile duct [16]. Resection may be a viable alternative for pCCA patients; however, their outcomes are poor with 5-year survival rates of only 10% [17]. Five-year survival rates for dCCA patients following resection are 23% [17]. iCCA has been further classified by the Liver Cancer Study Group of Japan (LCSGJ) into mass forming (MF-iCCA, the most common type, 60–80%), periductal–infiltrating (PI-iCCA, 15–35%), intraductal–growing (IG-iCCA, 8–29%), and an undefined and mixed subtype with a combination of hepatocellular cholangiocarcinoma (HC-CCA), which correlate to prognosis [7,18].

Moreover, eCCA and iCCA can be classified pathologically. iCCA is sub-classified into two main histological subtypes: (1) bile duct type (mucinous) arising from large bile duct consisting of larger tubules or papillary growth with taller columnar cells; (2) bile ductular type (mixed) deriving from small duct containing cuboidal cells forming smaller tubular or trabecular structures [1,18,19,20]. eCCA has a morphology of a typical nodular and papillary sclerosis among which the most common type is periductal [21]. Thus, eCCA histologically has a similar morphology as iCCA originating from the large bile duct [21].

Accumulating evidence indicates the potential roles of cancer stem cells (CSCs) in CCA carcinogenesis. Cardinale et al. [22] clearly proved that CSCs are largely and heterogeneously represented in CCAs, indicating CCA as stem cell-based cancer. CSCs are the subset of cancer cells which is capable of tumor initiation and malignant growth [23,24]. The role of CSCs in carcinogenesis is facilitated by the surrounding environment, i.e., tumor microenvironment (TME), and various mechanisms, such as epithelial to mesenchymal transition (EMT) and signaling pathways. Furthermore, therapeutic strategies for CCA are limited; thus, there is an urgent need for developing novel therapies. Since CSCs play important roles in carcinogenesis, targeting CSCs may represent a novel and promising therapeutic strategy. Therefore, this review discusses recent studies on the roles of CSCs in CCA and the possible therapeutic strategies targeting CSCs of CCA.

## 2. Characteristics of Cancer Stem Cells

The concept of CSCs proposes that there is a small population of CSCs in tumor tissues, which is capable of self-renewal and multilineage differentiation, and activates and sustains tumor growth [23,24,25]. Several studies have revealed that these cells play important roles in the generation of various solid tumors, including breast [23,26], ovarian [27], pancreas [24,28], brain [29], colorectal [30], lung [31], prostate cancers [32], osteosarcoma [33], melanoma [34], and HCC [35,36]. In CCA, Sell et al. [37] first proved the existence of stem cells using a chemically induced hepatocarcinogenetic rat model. In most solid cancers, CSCs represent <3% of the total cell mass, surprisingly in CCA, CSCs constitute >30% of the tumor mass, indicating the potential role of CSCs in CCA [22]. Several studies subsequently showed that the deregulated self-renewal of hepatic stem/progenitor cells represents as an early event in the carcinogenesis of CCA [38].

Furthermore, CSCs are highly aggressive during oncogenesis and responsible for resistance to chemotherapy and radiation therapy and tumor recurrence [39,40,41,42]. Intriguingly, the CSC profile of mucinous iCCA was similar to that of pCCA [22]. The CSCs in CCA are derived from the ductules and/or canals of Hering, where hepatic stem cells are situated [43,44]. However, the exact mechanism of genesis of CSCs remains obscure and the process of CSC formation is still not completely understood.

## 3. Surface Markers of Cancer Stem Cells

Surface markers are important for identifying and isolating CSCs, including CCA stem cells. Furthermore, they could act as the potential therapeutic markers of CCA. However, CSC markers for CCA have not been extensively studied. Immunohistochemical examination revealed a number of CCA CSC markers, such as CD133, CD24, CD44, EpCAM, Sox2, CD49f, and CD117, which have been described in detail in the following sections and Table 1. Interplay between the surface markers and signaling pathways was also discussed. Oncofetal markers are detected in both hepatoblasts/hepatic progenitor cells and liver CSCs when initiating signaling pathways during cancer progression [45]. It is already known that HCC initiation is prompted by CSCs [45]; however, limited research is dedicated to CCA in this aspect.

### 3.1. CD133

CD133, also known as prominin-1, is an important marker of CSC niches in several solid tumors [33], including CCA [47,72,73] and HCC [35,74,75]. However, the results for CCA are still controversial. Shimada et al. [47] first identified CD133 as a potential prognostic indicator of iCCA. In their study, CD133 was demonstrated to present more frequently in intrahepatic metastatic tissues and correlate with the expression of hypoxia-inducible factor-1α. The 5-year survival rate was much lower in the CD133+ patients than in CD133- patients (8.0% vs. 57.0%) [47]. This finding was further confirmed by Leelawat et al. [46], who reported a prominent expression of CD133 in CCA specimens (67.6%) that was significantly associated with metastasis of the lymph nodes and positive surgical margins. Moreover, CD133 has been reported to be associated with inflammation-related DNA damage [48]. Higher expression levels of stem/progenitor cell factors, such as Bmi1, 8-nitroguanine, DNA damage response (DDR) proteins (phosphorylated ATM and γ-H2AX), and manganese-SOD were higher in CD133+ tumor tissues than in CD133- tumor tissues.

On the other hand, Fan et al. [72] revealed the opposite results to those obtained by Shimada et al. The median survival time was shorter for the CD133- patients than that for CD133+ patients (4 months vs. 14 months), indicating association of better prognosis with CD133 expression.

However, there was a recent study by Cai et al. [49] demonstrating that the CD133+ non-mucin producing iCCA patients had significantly higher metastasis rate (36.7% vs. 10.1%) and shorter overall and disease-free survival time than CD133- patients. In addition, a higher number of CD133+ patients exhibited cancer recurrence than CD133- patients (90.9% vs. 64.3%), and CD133 might be associated with transforming growth factor-β (TGF-β)-related EMT alterations. Therefore, most studies support the concept that CD133 is associated with poor prognosis and might be a potential prognostic marker for CCA.

### 3.2. CD24

CD24, a membrane sialoglycoprotein also known as heat-stable antigen (HSA), is present in several solid tumors [76,77,78]. Similarly, CD24 expression has been reported in CCA and is found to be related to the clinicopathological data in many studies. However, there were no further studies after Leelawat’s study [53]. CD24 was expressed in ~51–81.8% of the CCA patients with a significantly shorter survival time [50,51]. Furthermore, the CD24 expression was associated with a lower median survival time in patients receiving chemotherapy and radiation therapy [51]. In addition, CD24 expression was significantly associated with lymph node metastasis and overall survival [52,79]. Intriguingly, the correlation of CD24 and matrix metalloproteinase-7 (MMP-7) has been observed [52]. Leelawat et al. [53] further elucidated the mechanism involving CD24 and discovered the association between CD24 and CXC chemokine receptor 4 (CXCR4) and its correlation with cell invasion. Activation of extracellular signal-regulated kinase (ERK) 1/2, which is a downstream molecule of the CXCR4 signaling pathway was also correlated with CD24. These results suggested that activation of the mitogen-activated protein kinase (MAPK)/ERK pathway might be the possible underlying mechanism for CD24-mediated cell invasiveness [52,53]. Importantly, CD24 was not detected in normal or inflamed epithelium, suggesting that CD24 might be a useful biomarker for early CCA detection [80] and a therapeutic target for CCA as elucidated below.

### 3.3. CD44

CD44, a transmembrane glycoprotein receptor for hyaluronic acid, plays important roles in cell migration, differentiation, and survival signaling in both normal stem cells and CSCs [81,82]. In addition, Kunlabut et al. [54] reported that CD44 was expressed in normal biliary cells adjacent to tumor areas, indicating the CD44 is critical in the early stages of carcinogenesis. Gu and Jang [55] further revealed that CD44 expression was associated with periductal infiltrative type, poor differentiation, and vascular invasion. Morine et al. [57] further demonstrated that CD44+ iCCA patients exhibited a worse prognosis than the CD44- iCCA patients in terms of 5-year survival (19.3% vs. 55.5%), indicating that CD44 is an important marker and prognostic indicator for iCCA.

Moreover, CD44v (the variant isoform) was recently discovered to be an important CSC marker; it was shown to regulate reactive oxygen species (ROS) defense system by stabilizing xCT (a cysteine–glutamate transporter) and upregulating the glutathione level resulting in cancer development and chemotherapy resistance [56]. In particular, CD44 variant 9 (CD44v9) is associated with chronic inflammation-induced cancer [58]. Higher CD44v9 expression was demonstrated in human liver fluke *Opisthorchis viverrini*-related CCA (OV-CCA) tissues than in non-OV-CCA tissues. Furthermore, CD44v9 expression was correlated with the expression of inflammation-related markers, S100 calcium-binding protein P (S100P) and cyclooxygenase-2 (COX-2), suggesting that CD44v9 might be a novel CCA stem cell marker and may be involved in inflammation-related cancer progression [58]. Therefore, CD44 might be a promising therapeutic target for CCA.

### 3.4. Epithelial Cell Adhesion Molecule (EpCAM)

Epithelial cell adhesion molecule (EpCAM) is a hemophilic, Ca^2+^-independent cell–cell adhesion molecule expressed in several human epithelial tissues. EpCAM is emerging as a specific CSC marker generally occurring at an early stage of neoplastic transformation, and it is associated with cell expansion and poor prognosis [83,84,85]. However, there have not been many studies on CCA. EpCAM is not expressed in adult liver, whereas it is expressed in the majority of hepatocytes of the embryonic liver, indicating its self-renewal and differentiation potential [86]. Sulpice et al. [59] identified that the EpCAM gene is upregulated in CCA and demonstrated that the overexpression of EpCAM in the stroma of CCA correlated with poor prognosis and disease-free survival. A recent study conducted by Julich-Haertel et al. [60] with a large sample size (172 HCC or CCA patients) revealed that the abundance of AnnexinV^+^ EpCAM^+^ CD147^+^ tumor-associated microparticles (taMPs) was elevated in HCC and CCA, indicating that they are a novel biomarker of HCC and CCA.

### 3.5. Sex Determining Region Y-box 2 (SOX2)

SOX2 is another potential biomarker for CCA which is an important transcriptional regulator in sustaining regeneration for embryonic stem cells [87]. SOX2 is critical in carcinogenesis and tumor progression in a number of cancers, such as neuroblastoma and testicular germ cell tumor [88,89]. In CCA, overexpression of SOX2 correlated with increased cell proliferation, suppressed cell apoptosis, aggressive behavior of enhanced cell migration, and invasion and poor overall survival [61]. In addition, SOX2 was proved to be highly associated with lymph node metastasis and worse overall survival [55]. There were no more studies on SOX2 in CCA.

### 3.6. CD49f

CD49f, which is also known as integrin α6, is found in adult stem cells [90]. Furthermore, CD49f plays an important role in the generation of some solid tumors, including osteosarcoma and hemangioma [91,92]. In CCA, Ding et al. [62] reported that iCCA tissues exhibited higher expression of CD49f than non-tumor samples. CD49f may enhance cell proliferation via ERK/AKT pathways and was associated with a migratory and invasive phenotype of iCCA cells and lower postoperative 5-year overall survival (OS) rate. Cavalloni et al. [63] established and characterized an Italian iCCA cell line, MT-CHC01, which expressed high levels of CD49f (98%). Thus, CD49f might be a promising biomarker for prognosis and targeted therapy.

### 3.7. CD117

CD117, also known as c-kit, is a transmembrane tyrosine kinase receptor found in hematopoietic progenitor cells. Although the significance of CD117 expression in hepatic progenitor cells is not conclusive, that high CD117 expression (83.3%) has been reported in combined HC-CCA [64]. However, a recent study by Xu et al. [65] confirmed that NCAM+ c-Kit+ iCCA RBE cells were highly proliferative and tumorigenic compared with NCAM- c-Kit- iCCA RBE cells. This study indicated that NCAM and c-Kit might be important markers for iCCA CSCs and potential CCA therapeutic targets [65].

### 3.8. Stem Cell Factor (SCF)

SCF is the ligand of the c-kit receptor and mediates cell survival, migration, and proliferation. It a hematopoietic factor that induces stem cell maturation and differentiation [93]. There have been limited studies on SCF in CCA. Mansuroglu et al. [66] discovered that SCF is expressed in various cell populations, proliferating biliary cells, macrophages, and liver myofibroblasts, and c-kit is presented on hepatocytes of the regenerating nodules and proliferating bile ducts of CCA during cholangiocarcinogenesis. This indicated that the SCF-c-kit system might contribute to tumor progression and could be used for early prognosis and targeted therapy.

### 3.9. Sal-Like Protein 4 (SALL4)

A novel stem cell marker, SALL4, is a member of a family of zinc finger transcription factors, expressed in embryonic stem cells and hematopoietic stem cells. Moreover, SALL4 is expressed in several solid tumors, including CCA and hematopoietic tumors [67,94,95]. SALL4 is crucial for cell proliferation and sustaining self-renewal by interacting with Oct3/4, Sox2, and NANOG [96]. A more recent investigation showed that cholangiolocellular carcinoma (CLC), a stem-cell subclass of mixed HC-CCA, were characterized as SALL4+, which was associated with poor clinical outcome [97]. These studies suggested that SALL4 might be a novel therapeutic target for CCA.

### 3.10. CD147

The role of CD147 was recently identified in CCA [68]. CD147 or extracellular matrix metalloproteinase inducer (EMMPRIN), is a transmembrane protein that can induce matrix metalloproteinases (MMPs). CD147 expression has been associated with cell migration, invasion, and metastasis as evident by an increase in F-actin rearrangement. The underlying mechanisms included the activation of MMP-2 activity and enhancement of EMT as shown by an elevated level of mesenchymal markers, such as Slug, vimentin, and N-cadherin, and suppression in levels of epithelial markers, such as E-cadherin and claudin-1, and the adhesion molecule, ICAM-1. These results highlighted the critical role of CD147 in CCA metastasis and indicated CD147 as a potential therapeutic target for CCA.

### 3.11. Stem Cell Antigen 1 (Sca-1)

Stem cell antigen 1 (Sca-1) is a phosphatidylinositol anchored protein belonging to the Ly-6 antigen family. Sca-1+ prostate cells exhibit multiple stem/progenitor cell properties [98]. Furthermore, Sca-1 was expressed in hepatic progenitor cells and was related to a significant increase in proliferation via epidermal growth factor (EGF) stimulation concomitant with activation of the phosphorylation of ERK1/2 and Cyclin D1. Moreover, the Wnt/β-Catenin pathway can act synergistically with EGF to significantly enhance hepatic progenitor cell (HPC) colony formation [69].

### 3.12. Laminin-332

Laminin-332, which is a large family of extracellular matrix proteins, is formed by three subunits (α, β, and γ), and promotes tumor progression and dissemination. In CCA, laminin-332 expression, particularly that of its γ2-chain, is essential for sustaining the self-renewal of CSCs and is responsible for resistance to doxorubicin and sorafenib, which was mediated by mammalian target of rapamycin (mTOR) activation. Laminin-332 increased K19 expression, phosphorylated mTOR, and decreased phospho-histone H3 expression, leading to reduced cell mitosis [70].

### 3.13. Aldehyde Dehydrogenase (ALDH)

Shuang et al. [71] reported that transforming growth factor-β1 (TGF-β1)-induced EMT provides CCA cell line, TFK-1, with stem cell-like features, such as CSC biomarker aldehyde dehydrogenase (ALDH), and enhanced resistance to chemotherapeutic drugs, 5-fluorouracil. Furthermore, CCA cells with the expression of ALDH displayed decreased E-cadherin expression, and upregulation of vimentin, fibronectin and N-cadherin, in comparison with ALDH^-^ cells [71]. Such an intimate relationship between EMT and stemness may play a critical role in promoting metastasis. The concept that only metastasizing cells with self-renewal features are capable of tumor dissemination [99] is emerging. ALDH+ cells isolated from TFK-1 cells displayed increased proliferation potential in vitro and tumourigenic ability in vivo. Furthermore, TGF-β1 and ALDH1 expression were correlated with poor prognosis in patients with CCA.

## 4. Tumor Microenvironment (TME)

**Figure 1:**
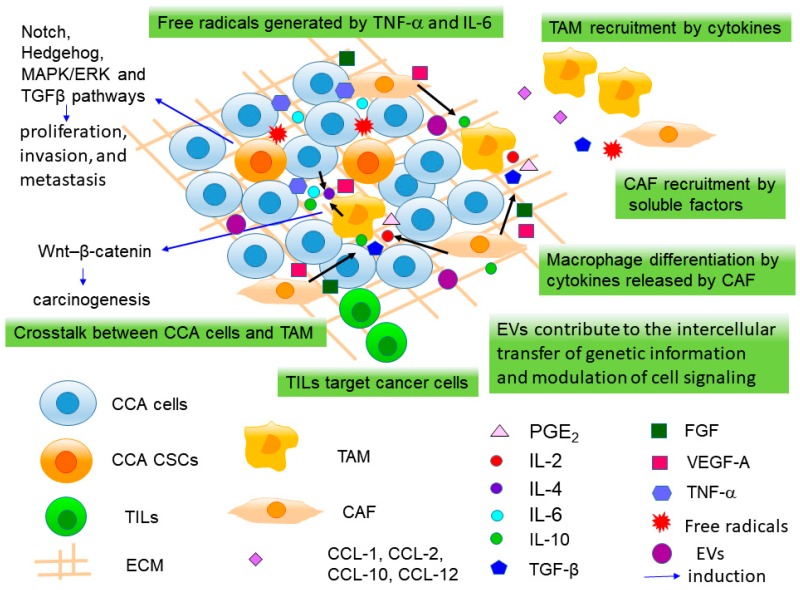
The tumor microenvironment (TME), ‘Cancer stem cell (CSC) niche’ of cholangiocarcinoma (CCA). The TME contains cancer-associated fibroblasts (CAFs), cancer cells/CSCs, tumor-associated macrophages (TAMs), tumor-infiltrating lymphocytes (TILs), and the extracellular matrix (ECM). TAMs are recruited into the TME by chemokines, MCP-1/CCL2, C-X-C motif chemokine ligand (CXCL)1, CXCL10 and SDF-1/CXCL12, secreted by tumor cells or other stromal cells. When infiltrating into TME, monocytes differentiate into M2 macrophages upon stimulation with soluble factors, prostaglandin E_2_ (PGE_2_), and cytokines, interleukin (IL)-2, IL-10 and transforming growth factor-β1 (TGF-β1) released by CAF and other inflammatory cells. In the crosstalk between TAMs and CCA cells, matrix metalloproteinases (MMPs), IL-4, IL-6, IL-10, vascular endothelial growth factor-A (VEGF-A), tumor necrosis factor-α (TNF-α) and TGF-β were secreted by lipopolysaccharide-activated TAMs. CAFs are recruited into the tumoral area and are activated by a variety of soluble mediators produced by both tumor cells, and the multiple inflammatory cells, such as platelet-derived growth factor (PDGF-D), TGF-β, reactive oxygen species (ROS) and FGF-2. CAF further recruit inflammatory cells, monocytes, macrophages, and endothelial cells to the tumor reactive stroma (TRS), through the secretion of VEGF, FGF, MCP-1/CCL2, SDF-1 and CXCL-14. TILs include CD8^+^ cytotoxic T lymphocytes, cytokine-secreting CD4^+^ T helper lymphocytes (Th), Forkhead box P3 (FoxP3)^+^ T leukocyte immunosuppressive regulators/regulatory T cells (Tregs), and B lymphocytes. TILs target cancer cells, and thus serve as a primary defence against cancer. T cell activation is tightly regulated by immune checkpoint pathways. TNF-α and IL-6 promote the generation of free radicals causing damage to DNA, resulting in genetic mutations and finally lead to tumor initiation. Wnt/β-catenin, Notch, Hedgehog, mitogen-activated protein kinase (MAPK)/extracellular signal-regulated kinase (ERK) and TGFβ pathways associated with cell growth dysregulation, invasion, and metastasis were activated surrounding CSCs. Extracellular vesicles (EVs) play as carriers for the intercellular transfer of genetic information and modulation of cell signaling of cancer cells.

CCA is characterized by a prominent desmoplastic stroma [100], formed by microenvironmental cells. The tumor microenvironment (TME) is critical in the regulation of tumor angiogenesis, invasion, and metastasis. It is made up of a biologically complex stroma composed of the cellular component of cancer-associated fibroblasts (CAFs), cancer cells/CSCs, tumor-associated macrophages (TAMs), tumor-infiltrating lymphocytes (TILs), vascular cells, and the extracellular matrix (ECM) [101] (Figure 1), and functions as a ‘CSC niche’. The CSC niche supports CSC proliferation and self-renewal and contributes to the maintenance of stemness and resistance to radiotherapy and chemotherapy [70,102,103,104,105,106].

The stroma of CCA tissue exhibits dramatic changes in its composition during pathogenesis of CCA with an upregulation of genes related to the cell cycle, ECM, and TGF-β pathway [107,108]. Stromal signature has been reported to be significantly correlated with worst prognosis, consistent with a role of TME in cholangiocarcinogenesis [108]. Animal models are essential techniques for cancer research; however, sometimes it can be challenging to study interactions between cancer cells and the stroma in mouse xenograft models since the TMEs are different between mice and human beings [6]. An orthotopic rat model developed by Sirica et al. [109], in which rat CCA cells were injected into the bile ducts of rats, thus the stroma and epithelial cells were derived from the same species. This animal model allows for investigating tumor–stroma interactions that more closely resemble those of patients.

### 4.1. Tumor-Associated Macrophages (TAMs)

TAMs are the major infiltrating immune cell population in the TME, and high tissue TAM density is correlated with poor prognosis of CCA patients [110,111]. TAM, which are derived from circulating CD14^+^/CD16^+^ monocytes, are recruited into the TME by a wide range of chemokines, including monocyte chemoattractant protein (MCP)-1/CCL2, C-X-C motif chemokine ligand (CXCL)1, CXCL10, and stromal-derived factor (SDF)-1/CXCL12, secreted by tumor cells or other stromal cells [112,113] (Figure 1). When infiltrating into TME, monocytes differentiate into M2 macrophages (alternatively activated macrophages) upon stimulation with soluble factors, such as prostaglandin E_2_ (PGE_2_), and cytokines, such as interleukin-2 (IL-2), IL-10, and TGF-β1 released by CAF and other inflammatory cells [114] (Figure 1). Thanee et al. utilized an Ov-induced hamster CCA model, and demonstrated that alteration of TAMs is a characteristic of early CCA and TAMs play key roles in progression and metastasis of CCA [115].

Several molecules secreted by lipopolysaccharide-activated TAMs, such as MMPs, IL-1, IL-4, IL-6, IL-8, IL-10, vascular endothelial growth factor-A (VEGF-A), tumor necrosis factor-α (TNF-α), TGF-β, colony stimulating factor (CSF-1), granulocyte-macrophage colony stimulating factor (GM-CSF), fibroblast growth factor (FGF)-1/2, platelet-derived growth factor (PDGF), insulin-like growth factor (IGF)-1, leukemia inhibitory factor (LIF) and prostaglandins, interferon (IFN)-γ, which stimulate EMT, tumor growth, invasion and metastasis, and have been reported to be involved in crosstalk between TAMs and CCA cells [111,114,116,117,118]; however, the Wnt pathway is the most extensively investigated pathway so far. TAMs induce canonical Wnt–β-catenin signaling pathway in CCA cells via Wnt ligands (Wnt3a and Wnt7b), thereby participating in cholangiocarcinogenesis [118,119]. In CCA, WNT7B was highly expressed in CD68^+^ TAMs, similarly to that revealed by Boulter et al. [119] in a mouse xenograft model of CCA. In this model, macrophage depletion reduced pro-proliferation genes, *BIRC5*, *CCND2* and *CCNE*, as well as an increase of pro-apoptotic genes, *BAX1*, resulting in reduced proliferation and induced apoptosis [119].

Recently, Raggi et al. [106] identified several secreted factors, IL-13, IL-34, and osteoactivin, from CCA spheres that promoted TAM-like phenotype. This finding presents a novel mechanism in which CCA CSCs activate TAMs that promote CCA development [106]. Likewise, other critical ECM remodeling-related genes, particularly metalloproteinase ADAM10, ADAM17, and MMP2, are largely expressed by CSC-associated TAMs. Importantly, TAMs associated with the CSC niche display an unique feature, a mixed M1/M2 phenotype, enhanced adhesive and invasive capabilities in vitro, and increased tumor-promoting functions in vivo. Their findings also indicate that in CCA, TAM-derived CCL18 and CXCL9 may direct the self-renewal and drug-resistance of CSC [106]. However, this work was conducted using CCA sphere medium; thus, more work is required to specifically determine the factors secreted by CSCs. Furthermore, it was reported that periostin, a disulfide-linked cell adhesion protein, plays an important role in tumor progression [120]. Recently, it was proven that periostin is secreted by CD44^+^ iCCA stem cells, and may serve as a chemoattractant for M2 TAM recruitment [121].

### 4.2. Cancer-Associated Fibroblasts (CAFs)

CAFs are the major stromal cell population in the CCA TME [122], they are activated myofibroblasts, identified by the expression of α-smooth muscle actin (α-SMA) [122,123]. In vitro and in vivo evidence demonstrated the role of α-SMA^+^ CAFs in CCA development and drug resistance; inducing CAF apoptosis reduces cancer cells and metastasis in a syngeneic rat CCA model [124]. The strengths of the syngeneic orthotopic rat model include the presence of a TME and an immunocompetent host. However, the limitation is abdominal manipulation which may lead to surgical risk to animals. Another disadvantage is the absence of de novo CCA development [125]. Clinical studies indicated the stromal expression of α-SMA was correlated with poor overall and disease-free survivals, and thus has the strong prognostic significance [126], particularly in eCCA [127]. However, CAFs’ cell source remains unclear in CCA, they may derive from quiescent hepatic stellate cells (HSCs), portal fibroblasts, as well as circulating bone marrow-derived mesenchymal cells [128,129]. CAFs are recruited into the tumoral area and are persistently activated by a variety of soluble mediators produced by both tumor cells, and multiple inflammatory cells. Among these factors, PDGF-D, TGF-β, reactive oxygen species (ROS) by secretion of nitric oxide (NO) and FGF-2 are more extensively investigated [130]. When close to transformed cells in tumoral ducts, CAF support tumor growth by overexpression of hepatocyte growth factor (HGF), heparin-binding epidermal growth factor (HB-EGF) [131], TGF-β and SDF-1/CXCL12 [114]. Indeed, HGF, HB-EGF and SDF-1 stimulate CCA cells to migrate through activating ERK-1/2 and Akt [6]. On the other hand, CAF contribute to further recruit inflammatory cells, monocytes, macrophages, and endothelial cells to the tumor reactive stroma (TRS), through the secretion of a number of growth factors, such as VEGF and FGF, as well as a vast number of cytokines and chemokines, including MCP-1/CCL2, SDF-1 and CXCL-14.

Furthermore, CAFs are capable of stimulating tumor invasion by inducing structural changes in the ECM, including stimulation of neuropilin-1, a co-receptor and signaling amplifier of a variety of VEGF family proteins, MMP-1, MMP-2 and MMP-9, promoting matrix degradation, periostin and tenascin C [132,133]. Periostin and tenascin C, which are highly reactive ECM components, activate integrins α5β1 and α6β4, which are transmembrane heterodimeric receptors regulating cell–cell and cell–ECM interactions [132]. Their upregulation leading to upregulation of the phosphoinositide 3-kinase (PI3K)/AKT signaling pathway, finally induces escape from apoptosis and cancer cell invasion. The ability of CAFs of promoting CCA cell invasion was confirmed in a syngeneic rat model, in which CAF depletion led to tumor growth inhibition and thus prolonged host survival [124]. Also, CAF may be involved in chemoresistance of CCA cells by activating periostin, PGE_2_, sphingosine-1-phosphate and PDGF-B. In this aspect, ECM, which acts as a reservoir for these soluble factors, may further support these CAF-mediated pro-tumorigenic effects [114].

### 4.3. Tumor-Infiltrating Lymphocytes (TILs)

TILs play an opposite role from TAMs and CAFs in TME. Convincing evidence in both mouse models and human patient samples demonstrated the role of the adaptive immune system is targeting cancer cells, and thus serve as a primary defence against cancer [134]. TILs are present in many solid tumors and highly heterogeneous including CD8^+^ cytotoxic T lymphocytes, cytokine-secreting CD4^+^ T helper lymphocytes (Th), Forkhead box P3 (FoxP3)^+^ T leukocyte immunosuppressive regulators/regulatory T cells (Tregs), and B lymphocytes [135]. CD8^+^ cytotoxic T cells are able to recognize tumor antigens, and kill cancer cells. CD4^+^ Th recognize tumor antigens that can assist CD8^+^ T cells, macrophages to phagocytose tumor cells, and B cells to produce antibodies against tumor cells. CD4^+^ TILs locate mostly in the peritumoral region [136], whereas CD8^+^ TILs prevail in the intratumoral CCA tissue [136,137]. A number of studies confirm that increased CD4^+^ and CD8^+^ TILs in CCA and extrahepatic biliary tract cancers (BTCs) are correlated with reduced lymph node metastases, decreased venous and perineural invasion and better overall survival [137,138,139]; consistently low CD8^+^ TILs are correlated with poor overall survival [140].

T cell activation is tightly regulated by two types of ‘immune checkpoint pathways’, which either co-stimulate or co-inhibit T cells. T cells infiltrating into TME generally have high expression of co-inhibitory receptors, whereas tumor cells and intra-tumoral antigen-presenting cells can express ligands for these co-inhibitory receptors [141,142]. By investigating the CCA tissues, reduced cytotoxic immune cells and increased Treg together with the activation of two co-inhibitory receptors programmed death-1 (PD-1) and cytotoxic T lymphocyte antigen-4 (CTLA-4) on tumor-infiltrating T cells and their ligands on cancer cells indicate that immunosuppression within the TME that facilitates tumor recurrence in CCA [143,144]. It has been shown that programmed death ligand-1 (PD-L1), one of the PD-1 ligands, binds to PD-1 of the T cells and initiates apoptosis of a tumor-specific T-cell leading to impair T-cell-mediated antitumor immune responses, and local immunosuppression, thus promoting tumor growth and metastasis [145,146,147]. It has been demonstrated that high expression of CTLA-4 and PD-L1 were correlated with the worse prognosis of CCA [148,149,150]. A recent study by Zhu et al. [151] showed that tumor PD-L1 overexpression was associated with activated CD8^+^ T-cells in iCCA, and was significantly associated with superior overall survival. Hence, these molecules represent promising therapeutic targets for immune therapies for CCA patients.

In addition, tumor-infiltrating T cells, triggering by CCL2 produced by cancer cells, TAMs, and CAFs acquire CD4/CD25 expression and transform to Treg in TME [152]. Within tumors, Tregs secret TGF-β and IL10, which leads to an immunosuppressive environment by accelerating the death of cytotoxic T cells and natural killer cells. Moreover, Tregs also bind to IL2, leading to its depletion in TME and thus inhibiting the activation of additional immune cells [153].

Even though several studies have drawn attention to T cells in CCA, the studies on B lymphocytes are lacking. B cells have been identified in TIL populations in CCA, and high density of CD20^+^ B lymphocytes were correlated with a favorable overall survival [136,137]. However, no data revealing their role in CCA pathogenesis are currently available and future studies are required to clarify this.

### 4.4. Other Factors

In addition to various cell types, soluble factors, such as cytokines, chemokines, growth factors, morphogens, and proteinases, which are secreted by cancer cells and stromal cells, form another important component of a pro-inflammatory TME [101]. The proinflammatory TME can induce myofibroblast activation, CSC initiation, and recruitment of a variety of inflammatory cell types [101]. The proinflammatory cytokines, such as TNF-α and IL-6, promote the generation of free radicals causing damage to DNA, that cause genetic mutations and finally lead to tumor initiation. Tumor growth is also facilitated by proinflammatory cytokines that stimulate cell proliferation and decrease apoptosis. On the other hand, anti-inflammatory cytokines, such as IL-10 and TGF-β, are involved in tumor evasion and invasion by activating the EMT [80].

Apart from the soluble factors secreted by cells in the TME, a number of specific pathways associated with cell growth dysregulation, invasion, and metastasis were activated surrounding CSCs in the TME [80], such as Wnt/β-catenin, Notch, Hedgehog, MAPK/ERK, and TGFβ pathways [2,154] (Figure 1). This indicated that the interplay between CSC, inflammatory components, and TME is crucial in carcinogenesis of CCA [80]. The Wnt/β-catenin pathway, containing Wnt2, Wnt3, β-catenin, and transcription factor 4, and its target genes, *c-myc* and *cyclin D1*, is critical in cell proliferation and cell apoptosis [155,156]. Dysregulation of this pathway encourage the hepatic stem/progenitor cells to self-renew and occurs at the early stage of carcinogenesis [38,119]. Notch signaling pathway sustains the hepatic progenitor cells [157] and aberrant expression of Notch receptors 1 and 4 may be critical during iCCA progression [158]. Inhibiting this pathway resulted in downregulation of cyclin E expression and induction of TNF-related apoptosis in CCA [159]. Activation of the Hedgehog pathway by stromal cells is required for the proliferation, migration, and invasion of CCA cells and promotes the hepatic stem cell proliferation [160]. In addition, the MAPK/ERK pathway is required for the proliferation of hepatic stem cells [161,162].

Furthermore, extracellular vesicles (EVs), i.e., microvesicles and exosomes, are increasingly being recognized to play an important role as carriers contributing to the intercellular transfer of genetic information and modulation of cell signaling of cancer cells in TME [163] (Figure 1). The presence of microRNA-laden extracellular vesicles in human bile has been reported in CCA patients [164,165]. Moreover, EpCAM and CD133 are expressed by microparticles, AnnexinV^+^ EpCAM^+^ CD147^+^ and AnnexinV^+^ EpCAM^+^ ASGPR1^+^ CD133^+^ tumor-associated microparticles (taMPs), in CCA liquid biopsy and have been proved to be a significant non-invasive diagnostic and prognostic tool [60]. CCA-cell-derived EVs can generate tumor stroma by modulating fibroblastic differentiation of mesenchymal stem cells (MSCs) and releasing proinflammatory factors, such as IL-6, which lead to CCA proliferation [166].

Since CSCs play a critical role in maintaining and promoting a pro-inflammatory TME and in cancer initiation and progression, these cells are promising targets for normalizing TME. In particular, CSCs could be dedifferentiated to normal epithelial cells by the mesenchymal–epithelial transition (MET) approach [167]. Moreover, differentiation of CSCs may be another viable approach. As a result, eliminating the CSC niche or suppressing the CSC niche formation may lead to normalization of TME.

## 5. Epithelial-to-Mesenchymal Transition (EMT)

Metastasis is a critical factor in poor prognosis of the patients with CCA [17,168]. The EMT process involves profound phenotypic changes including the following four steps: (1) Reducing cell-cell contacts; (2) acquisition of migratory and invasive properties to invade the surrounding stroma; (3) dissemination to distant organs through the lymphatic and/or hematogenous circulation; (4) Engraftment at the distant sites [169,170]. Increasing evidences demonstrated EMT is an important mechanism involved in invasiveness, metastasis and chemoresistance of cancer [169,171,172]. Furthermore, it has been demonstrated that TGF-β1-induced EMT plays a critical role in generating CSCs [71], and is involved in maintaining CSC properties, such as self-renewal and differentiation [173]. Most CCA CSCs possess both epithelial and mesenchymal features and express EMT markers [22,174]. TGF-β1 induces EMT-mediated cancer progression with mesenchymal features characterized by reduced epithelial cadherin (E-cadherin) and cytokeratin 19 expression and increase in expression of mesenchymal markers, such as vimentin and S100A4, via the Snail transcription factor, leading to activating collagen type I and MMP2, and the likelihood of lymph node metastasis and a poor survival rate. Fabris et al. [175] revealed that nuclear expression of S100A4 calcium-binding protein displayed increased CCA invasiveness and metastasization when xenotransplanted into severe combined immunedeficiency (SCID) mice. The pathogenesis of S100A4 was further supported by in vitro studies in which down-regulation of nuclear S100A4 in CCA cells significantly decreased their motility and invasiveness. Therefore, EMT, activated by TGF-β1/Snail, is closely associated with the invasiveness of CCA [71,176,177,178]. Interestingly, TNF-α can induce EMT of CCA cells via TGF-β resulting in activation of EMT-related proteins, ZEB2 and S100A4 [179,180]. In CCA cells, TNF-α and IFN-γ promote the expression of chemokine receptors particularly CCR5 and induce the production of CCL5 in MSCs. The CCL5/CCR5 axis induces CCA metastasis and growth via Akt/NF-κB signalling enhancing the expression of MMP [181].

Upon lipopolysaccharide stimulation, macrophages elicit EMT-like phenotypic changes in CCA cells via the TNF-α activation of Snail and ZEB2 [117,179,180]. Human CCA cells harvested from activated macrophages and cultured with conditioned media actually showed decreased E-cadherin and K-19, associated with increased S100A4 and MMP-9 [117], and revealed increased migration in vitro [115]. Similarly, SDF-1 produced by CAFs, was demonstrated to induce the invasiveness of cultured CCA cells, associated with de novo expression of vimentin, and reduction of E-cadherin and membranous β-catenin [182]. Interestingly, an in vivo xenotransplant severe combined immunodeficiency (SCID) male mouse model with CCA cells proved that CAFs are not generated through an EMT, but rather their recruitment was regulated via PDGF-D secreted by CCA cells. PDGF-D promotes fibroblast migration by binding to its cognate receptor PDGFRβ, and activates its downstream effectors, Rho GTPase and c-Jun N-terminal kinase (JNK) [128].

## 6. Therapeutic Implications

### 6.1. Targeted Therapies

Since it is known that CSCs play a significant role in carcinogenesis of CCA, therapeutic strategies can target the surface markers and signaling pathways of CCA CSCs and proteins involved in TME and carcinogenesis as described above. Several targeted therapies have been demonstrated in in vitro and in vivo experiments (Table 2). However, no relevant clinical trials have been conducted so far. Inhibiting CD133 by a murine anti-human CD133 antibody conjugated to a potent cytotoxic drug, monomethyl auristatin F (AC133-vcMMAF), reduced the growth rate of Hep3B hepatocellular cells in vitro with IC_50_ of 2–7 ng/mL and induced apoptosis. AC133-vcMMAF inhibited in vivo tumor growth in SCID mice [183]. EpCAM inhibition by RNA interference in hepatic stem/progenitor cells resulted in a decrease in tumorigenicity and invasiveness [184,185]. Moreover, CD44 silencing by siRNA suppressed aggressiveness, migration, and adhesion in cholangiocarcinoma cell lines [186]. In addition, CD24 inhibition significantly reduced the invasion of RMCCA1 CCA cells [52]. Further study of CD24+ cells by Leelawat et al. [53] revealed that CXCR4 activity was inhibited by AMD3100, a non-competitive antagonist of CXCR4. AMD3100 significantly affected the cell motility and invasion of CD24+ cells [53]. AMD3100 also abolished the CXCL12-induced phosphorylation of mitogen-activated protein kinase (MEK) 1/2 in CCA cells [187]. A similar study by Leelawat et al. [53] showed that U0126 (a MEK/ERK inhibitor) significantly inhibited the motility of the CD24+ cells. Sulfasalazine, xCT inhibitor inhibited cell growth and induced autophagic cell death. Thus, an xCT-targeting drug may improve CCA treatment by sensitizing CCA cells to chemotherapeutic drugs, such as gemcitabine, by inhibiting the cell’s ROS defensive system [56].

In addition, overexpression of the miRNAs, let-7c/miR-99a/miR-125b inhibited in vitro CCA mammosphere formation. It suggested that these miRNAs may be critical in the maintenance and proliferation of CCA CSCs, which further proved their potential to be used for developing a miRNA-based therapy for CCA [188]. Recently, Dana et al. [68] showed that inhibition of CD147 expression using siCD147 significantly reduced cell migration and invasion of CCA cells [68].

Furthermore, differentiation therapy forces cancer cells to resume differentiation into mature cells [194]. All-trans-retinoic acid (ATRA), the first differentiation agent, was found to be successful in treating acute promyelocytic leukemia [195]. An intimate relationship between CSCs and their niche is essential for sustaining drug resistance. Thus, disrupting the link between CSCs and their niche might be a potentially effective therapeutic approach involving reversing of the drug resistance of cancer cells [43].

Targeting CAFs from the tumor stroma is another possible therapeutic strategy. The cytotoxic drug, navitoclax, an inhibitor of Bcl-2, Bcl-X_L_, and Bcl-w, induced apoptosis only in CAFs in a syngeneic CCA rat model, with concomitant decrease in levels of desmoplastic ECM proteins and inhibition of tumor growth [124].

These results indicated that targeting surface CSC markers is a new and promising therapeutic strategy for CCA. However, more clinical trials are urgently needed for further validation.

### 6.2. Immune Therapies

Apart from targeted therapies, immune therapies have emerged as promising therapeutic strategies for several cancers. Immune checkpoints, CTLA­4 and PD­1, aim to maintain self-tolerance and prevent damage to normal tissue during an immune response. Cancer cells exploit several resistance mechanisms to evade immune surveillance, and thus, antitumor immune responses, including modulation of the local TME, creating an immunosuppressive milieu; downregulation of expression of immune checkpoint proteins and loss of MHC expression [196]. However, the exact mechanisms underlying the immune escape of CCA remain to be elucidated. CTLA-4 plays a crucial role in regulating T-cell tolerance and has become a main focus for immunotherapy [189]. Inhibitors and antibodies blocking the interactions between CTLA­4 or PD­1 and their cognate ligands, have demonstrated to be effective in various tumor types, with low immune­mediated toxicity [197], including HCC [190]. Both anti-CTLA-4 monoclonal antibodies and the PD-1 and PD-L1 inhibitors are clinically used for cancer immunotherapy (Table 2).

A higher expression of immune checkpoint molecules was detected in 45% of 260 biliary tract cancer patients [148]. In the studies involving smaller CCA sample sizes (*n* = ~54–99), PD­L1 expression was demonstrated in ~9–72% of specimens, and in ~46–63% of immune cells within the TME [150,198]. This expression of PD-L1 was significantly correlated with 60% reduction in overall survival compared to PD-L1 negative counterparts [150]. These results suggested that PD­1 or PD­L1 inhibitors might be effective for a substantial proportion of CCA tissue. There is limited data on clinical use of immune therapies for CCA. The anti-PD-1 antibody, pembrolizumab, is currently being used in phase I/II studies. Preliminary data revealed promising result in CCA with approximately 40% response rate. A phase II (NCT02628067) clinical trial is ongoing. The PD-L1 inhibitor, nivolumab, has just been approved for HCC but no corresponding data are available for CCA yet [189].

Moreover, a recent study by Zhou et al. [144] proved that inhibition of PD-1 or CTLA-4 as well as induction of tumor necrosis factor receptor superfamily member 18 (GITR) increases the *ex vivo* effector functions of tumor-infiltrating T cells from patients with CCA, indicating that these may be promising targets for immunotherapy. However, combination of immunotherapy with routine management might be required in order to promote the effector T cell penetrating from the tumor margin into the tumor bed [144]. A number of phase I and II trials are currently assessing the therapeutic efficacies of combination checkpoint inhibitor therapies in advanced BTC including combinations such as ipilimumab (CTLA4 inhibition) and nivolumab (PD1 inhibition) (NCT02834013, NCT02923934 and NCT03101566) or durvalumab (PDL1 inhibition) and tremelimumab (CTLA4 inhibition) (NCT02821754) and may maximize future therapeutic strategies [191] (Table 2).

The potential adverse effects should be considered when applying immune therapy for CCA. CCA patients with prevalent hepatic dysfunction and biliary obstruction are associated with high rates of adverse events in the study of cytotoxic therapies [199] raising the issue of an increased risk of immune­mediated hepatobiliary toxicity, such as cholestasis or hepatitis, when applying immune checkpoint inhibition [196]. Promisingly, El-Khoueiry et al. [190] demonstrated that the incidence of grade 3 or 4 treatment-related serious adverse events among 214 HCC patients in the phase I/II CheckMate 040 trial of PD­1 inhibitor, nivolumab, was approximately 4%, which is similar to the rates reported for other tumor types. In addition, autoimmune diseases, such as primary sclerosing cholangitis (PSC) and inflammatory bowel disease, which are also recognized as risk factors in a subset of CCA patients, raise another issue concerning the risk of flares when using immune therapies on this population [196]. It should be noted that patients with underlying autoimmune diseases were generally excluded from the clinical trials of immune therapies, consequently there are no data regarding the adverse effects of immune therapies in this subset of CCA patients [196].

### 6.3. Combination Therapies

Considering the extensive interplays between different cell types in TME and crosstalk between the various signaling pathways involved in cholangiocarcinogenesis, the development of combination therapies is inevitable. In particular, the domain of combination therapy should be pursued to develop a combination of targeted therapy and immunotherapy [196]. Xie et al. [192] developed a novel therapy combining nanotherapeutic blockade of CXCR4 by polymeric CXCR4 antagonist (PCX) with inhibition of hypoxia-inducible miR-210. In their study, combination PCX/anti-miR-210 nanoparticles resulted in significant CCA cell death through induction of apoptosis and reduced the number of cancer stem-like cells. Furthermore, the nanoparticles sensitized CCA cells to standard gemcitabine and cisplatin combination treatment by reversing hypoxia-induced drug resistance. The nanoparticles showed enhanced in vivo antitumor activity in a CCA xenograft model [192]. Therefore, combination PCX/anti-miR-210 nanoparticle + gemcitabine/cisplatin might be a promising combination therapy for CCA.

Moreover, Kawamoto et al. [193] demonstrated for the first time that metronidazole (MNZ) reduces cancer stemness by reducing ALDH activity and promoting MET, resulting decreased invasive potential and enhanced gemcitabine (GEM) chemosensitivity by enhancing equilibrative nucleoside transporter 1 (ENT1) and diminishing ribonucleotide reductase M1 (RRM1) in CCA cells. Therefore, this combination therapy has the potential to improve the prognosis of CCA patients [193].

As immunotherapy is emerging, there are trials investigating its combination with current first-line treatment strategies for advanced BTC including chemotherapy, transarterial catheter chemoembolization and radiofrequency ablation (NCT03111732, NCT03101566, NCT02821754) [191] (Table 2). The success of these trials will definitely provide more benefit for CCA patients and further help elucidate the possible underlying mechanisms of immune checkpoint signaling pathways and their interplay with other signaling pathways.

## 7. Conclusions and Future Directions

CCA is a highly aggressive and extraordinarily heterogeneous cancer; therefore, it is important to understand the mechanisms underlying its carcinogenesis. CSCs are critical in carcinogenesis, metastasis, chemoresistance, and recurrence for CCA. Studies applying animal models and human tissues have helped us further elucidate the interplay between CSCs and TAMs. However, compared with HCC, the studies on the role of CSCs in cholangiocarcinogenesis are relatively new, and thus data regarding this aspect are limited. Furthermore, there are no data currently available revealing the role of B cells in CCA pathogenesis and future studies are required to clarify this. Therefore, more studies on cholangiocarcinogenesis are needed to fully elucidate the whole picture.

Apart from the complexity of anatomical location, CCA exhibits a complicated pathogenesis, with interplays between cancer cells, CSCs, and the TME [174]. Thus, several different therapeutic strategies might be applied for CCA. Although targeted therapy is emerging as a promising and specific therapeutic strategy, it still harbors certain limitations. The markers and signaling pathways of CSCs are largely shared by normal stem cells, thereby limiting the specificity of targeted therapy to CSCs [41,200]. Further studies for identifying more CSC specific markers are necessary in order to prevent adverse effects to normal stem cells [43]. Furthermore, due to crosstalk of signaling pathways in CSC and between different cells in TME [200,201], it may be difficult to eliminate CSCs by targeting a single molecular marker or signaling pathway, and combination therapies are thus urgently needed for most cases.

Future research focusing optimized dosing and therapeutic regimens, and identifying more novel therapeutic targets for targeted, immunotherapy and combination therapies remains imperative. Future research effort should be focused on pushing the current novel and promising therapies into preclinical and clinical trials in order to develop novel therapeutic strategies for CCA, a highly invasive and chemoresistant tumor.

## Figures and Tables

**Table 1:** Surface Markers of Cancer Stem Cells in Cholangiocarcinoma (CCA).

Surface Markers	Functional Roles in CCA	Clinical Characteristics	References
CD133	metastasis of the lymph nodes; intrahepatic metastasis; inflammation-related DNA damage; cancer recurrence	poor prognosis; aggressive clinical features	[46,47,48,49]
CD24	tumor expansion; progression; lymph node metastasis; apoptosis	poor prognosis; shorter survival time; invasiveness; poor response to chemotherapy and radiation therapy	[50,51,52,53]
CD44/CD44v	tumor progression; metastasis; tumor relapse after treatment	shorter lifespan; poor prognosis; chemotherapy resistance	[54,55,56,57,58]
Epithelial cell adhesion molecule (EpCAM)	proliferation; recurrence; epithelial to mesenchymal transition	poor prognosis and disease-free survival	[59,60]
SOX2	increased cell proliferation, suppressed cell apoptosis, enhanced cell migration and invasion, lymph node metastasis	poor overall survival	[55,61]
CD49f	promote metastasis, invasion, and cell proliferation	poor prognosis	[62,63]
CD117	tumorigenesis, proliferation	poor prognosis	[64,65]
Stem cell factor (SCF)	tumor progression	poor prognosis	[66]
SALL4 (Sal-like protein 4)	proliferation	poor clinical outcome	[67]
CD147	cell migration, invasion, and metastasis	poor prognosis	[68]
Sca-1	proliferation	poor prognosis	[69]
Laminin-332	maintain self-renewal	chemotherapy resistance	[70]
Aldehyde dehydrogenase (ALDH)	proliferation, chemoresistance	poor prognosis	[71]

**Table 2:** The Potential Therapeutic Strategies for CSCs.

Therapeutic Strategies	Target	Mechanism	Treatment	References
Targeted therapies	CD133	suppressed tumor growth, induced apoptosis	anti-CD133-drug conjugate (AC133-vcMMAF)	[183]
	EpCAM	decreased cell number, tumorigenicity, spheroid formation and invasiveness	siRNA	[184,185]
	CD44	suppressed aggressiveness, migration and adhesion	siRNA	[186]
	CD44v	inhibited cell growth and activated cell death	cystine–glutamate transporter (xCT) inhibitor sulfasalazine	[56]
	CD24	reduced invasiveness	siRNA	[52]
	CD147	decreased cell migration and invasion	siCD147	[68]
	CXCR4	suppressed the motility of the CD24+ cells	AMD3100 (CXCR4 inhibitor)	[53]
	mitogen-activated protein kinase (MAPK)/extracellular signal-regulated kinase (ERK)	inhibited the motility of the CD24+ cells	U0126 (MEK/ERK inhibitor)	[53]
	IL-6/STAT3 signaling pathway	reduced mammosphere formation	let-7c/miR-99a/miR-125b	[188]
Immune therapies	cytotoxic T lymphocyte associated protein 4 (CTLA-4)	evaded immune surveillance: regulation of T-cell tolerance	anti-CTLA-4 monoclonal antibodies, ipilimumab	[144,189]
	programmed death 1 (PD-1) and programmed death ligand 1 (PD-L1)	evaded immune surveillance	anti-PD-1 antibody pembrolizumab; anti-PD-L1 inhibitor nivolumab	[144,189,190]
	CTLA-4 and PD-1	evaded immune surveillance	nivolumab and Ipilimumab (Phase II)	[191]
	CTLA-4 and PD-L1	evaded immune surveillance	durvalumab (PD-L1 inhibition) and tremelimumab (CTLA4 inhibition) (Phase I/II)	[191]
Combined therapies	chemokine receptor CXCR4 and hypoxia-inducible miR-210	inhibited cell migration; showed cytotoxic activity towards CCA cells and reduced the number of cancer stem-like cells; reversed hypoxia-induced drug resistance	combination PCX/anti-miR-210 nanoparticle	[192]
	Gemcitabine (GEM) and Metronidazole (MNZ)	suppressing ALDH activity, leading to decreased invasiveness and enhanced chemosensitivity	MNZ-induced mesenchymal–epithelial transition (MET) and enhancing chemosensitivity via increasing equilibrative nucleoside transporter 1 (ENT1) and reducing ribonucleotide reductase M1 (RRM1)	[193]
	Pembrolizumab + Capecitabine/Oxaliplatin	evaded immune surveillance; inhibited cell growth	immunotherapy + chemotherapy (Phase II)	[191]
	Nivolumab + Gemcitabine/Cisplatin or Ipilimumab	evaded immune surveillance; inhibited cell growth	immunotherapy + chemotherapy (Phase II)	[191]
	Durvalumab + Tremelimumab + TACE/RFA or Cryoablation	evaded immune surveillance; destruction of tumor	immunotherapy + radiofrequency ablation (Phase I/II)	[191]

## Abbreviations

CCA	Cholangiocarcinoma
iCCA	Intrahepatic cholangiocarcinoma
eCCA	Extra-hepatic cholangiocarcinoma
pCCA	Perihilar cholangiocarcinoma
CSC	Cancer stem cell
TME	Tumor microenvironment
EMT	Epithelial-to-mesenchymal transition
HCC	Hepatocellular carcinoma
LCSGJ	Liver Cancer Study Group of Japan
MF-iCCA	Mass forming intrahepatic cholangiocarcinoma
PI-iCCA	Periductal–infiltrating intrahepatic cholangiocarcinoma
IG-iCCA	Intraductal–growing intrahepatic cholangiocarcinoma
DDR	DNA damage response
TGF-β	Transforming growth factor-β
HSA	Heat-stable antigen
MMP-7	Matrix metalloproteinase-7
CXCR4	CXC chemokine receptor 4
ERK	Extracellular signal-regulated kinase
ROS	Reactive oxygen species
CD44v9	CD44 variant 9
OV-CCA	*Opisthorchis viverrini*-related cholangiocarcinoma
S100P	S100 calcium-binding protein P
CX-2	Cyclooxygenase-2
EpCAM	Epithelial cell adhesion molecule
TaMP	Tumor-associated microparticle
SOX2	Sex determining region ϒ-box 2
OS	Overall survival
SCF	Stem cell factor
SALL4	Sal-like protein 4
CLC	Cholangiolocellular carcinoma
EMMPRIN	Extracellular matrix metalloproteinase inducer
Sca-1	Stem cell antigen 1
EGF	Epidermal growth factor
CAF	Cancer-associated fibroblasts
TAM	Tumor-associated macrophages
ECM	Extracellular matrix
PGE_2_	Prostaglandin E_2_
IL	Interleukin
VEGF-A	Vascular endothelial growth factor-A
TNF-α	Tumor necrosis factor-α
EVs	Extracellular vesicles
PDGF	Platelet-derived growth factor
FAK	Focal adhesion kinase
SCID	Severe combined immunodeficiency
xCT	Cysteine–glutamate transporter
CTLA­4	Cytotoxic T­lymphocyte­associated antigen 4
PD-L1	Programmed death ligand 1
PD-1	Programmed death 1
TRS	Tumor reactive stroma
MCP	Monocyte chemoattractant protein
SDF	Stromal derived factor
Treg	T leukocyte immunosuppressive regulators
MNZ	Metronidazole
GEM	Gemcitabine
ALDH	Aldehyde dehydrogenase
FGF	Fbroblast growth factor
NO	nitric oxide
HGF	hepatocyte growth factor
HB-EGF	heparin-binding epidermal growth factor
FOX P3	Forkhead box P3
SCID	severe combined immunedeficiency
ENT1	equilibrative nucleoside transporter 1
RRM1	ribonucleotide reductase M1
